# Anomalous Development and Osseous Union of Three Teeth

**Published:** 1856-01

**Authors:** 


*Anomalous Development and Osseous Union of three Teeth.*—Dr. Colburn, dentist, of Newark, N. J., recently presented to the senior editor, for the Museum of the Baltimore College of Dental Surgery, a lower temporary incisor, or rather three incisors, having a complete osseous union throughout the entire length of two and to the crown of the third. Two of the teeth are very feebly developed, as well as the root of the third. The specimen, altogether, is not much larger than an ordinary temporary incisor. From between the crowns of two, the lingual surfaces of which are turned towards each other, the crown of the third emerges. The enamel of this last is somewhat eroded. The specimen presents a most singular example of anomalous development as well as osseous union, and the writer, in behalf of the faculty of the above institution, begs to thank Dr. C. for so interesting and valuable a contribution.

